# Postoperative Recovery in the Youngest: Beyond Technology

**DOI:** 10.3390/children11081021

**Published:** 2024-08-21

**Authors:** Carina Sjöberg, Mona Ringdal, Pether Jildenstål

**Affiliations:** 1 Department of Medicine and Health Sciences, Lund University, 223 62 Lund, Sweden; pether.jildenstal@vgregion.se; 2 Department of Anaesthesiology, Surgery and Intensive Care, Sahlgrenska University Hospital, 413 45 Gothenburg, Sweden; 3 Institute of Health and Care Sciences, Sahlgrenska Academy, University of Gothenburg, 413 46 Gothenburg, Sweden; mona.ringdal@fhs.gu.se; 4 Department of Anestesiology and Critical Care, West Hospital, 442 34 Kungälv, Sweden; 5 Department of Anaesthesiology and Intensive Care, Örebro University Hospital and School of Medical Sciences, Örebro University, 701 82 Örebro, Sweden

**Keywords:** clinical nursing research, patient safety, paediatrics, postoperative care, vital signs

## Abstract

Background: Measuring and interpreting vital signs in pediatric patients recovering from anaesthesia, particularly those up to 36 months old, is challenging. Nurses’ decision-making regarding the level of monitoring must balance patient safety with individualized care. This study aimed to explore the perceptions of critical care nurses and registered nurse anesthetists regarding their experiences and actions when making decisions about vital sign monitoring for children in post-anesthesia care units (PACUs). Methods: A qualitative study utilizing the critical incident technique was conducted. Interviews were performed with a purposeful sample of 17 critical care nurses and registered nurse anaesthetists from two hospitals. Results: Nurses reported that the rationale for decisions concerning the need for vital sign monitoring in children was both adequate and inadequate. Actions were taken to adjust the monitoring of vital signs, optimizing conditions for assessment and ensuring the child’s safe recovery. Conclusions: The complexity of accurately monitoring children makes it challenging for nurses in the PACU to adhere to guidelines. Evidence-based care and safety are compromised when technology has limitations and is not adapted for paediatric use, leading to a greater reliance on experience and clinical assessment. This reliance on experience is crucial for reliable assessment but also entails accepting greater risks.

## 1. Introduction

In the past 5 years, the population of very young children undergoing surgery has grown. In 2021, 15,281 surgical procedures for children up to 4 years old were performed in Swedish inpatient care, while the majority of surgical interventions (27,563) occurred on a routine, planned, and outpatient basis [1]. Enhancements in patient safety and the safe, effective management of paediatric airways significantly impact overall surgical safety and contribute to the increase in surgical procedures [2]. To enable secure recovery for patients in the post-anaesthesia care unit (PACU) after surgery and anaesthesia, nurses should be specially educated for the context due to the complexities of postoperative recovery [3]. However, there is no consensus regarding what formal personnel competence or education is needed to enhance safe care in the PACU—especially safe care for very young paediatric patients. Special knowledge is needed in several different domains, such as surgery procedures, anaesthesia methods, pharmacology, vital sign monitoring, and treatment for various emergent conditions [4].

The care required in the PACU is similar to intraoperative anaesthesia care; the conditions required to handle complex situations are high-quality teamwork and non-technical skills such as communication, leadership, and shared situational awareness [5]. In the PACU, vital signs including the respiratory rate, heart rate, blood pressure, temperature and pain level indicate the child’s current postoperative physiological status. To predict and prevent clinical deterioration, observing and assessing vital signs are crucial for the nurses and their decision-making [6]. After procedures requiring intravenous or inhaled anaesthesia, children’s vital signs are recorded frequently to monitor physiological stability.

The frequency of postoperative vital sign measurement in every institution typically follows the guidelines issued by the ASA [7], or the ESAIC [8]. These guidelines are based on factors such as anaesthesia type, surgical site, clinical status, co-morbidities, and the health care provider’s orders [7,8]. Since the guidelines are recommendations rather than established care standards, individual institutions determine their own population-specific departmental policies and protocols.

The optimal way to perform monitoring for children after anaesthesia in the PACU is to provide an environment that is adapted to the child’s needs and prepared for critical care. This is because there is potential for rapid physiological deterioration to occur at any time as children emerge from general anaesthesia [7]. In European PACUs, anaesthesiologists are most commonly responsible for the medical care provided, working in teams alongside nurses. The education level of the nurses usually varies, but it is common to have specially trained nurses, including educated anaesthesia or intensive care nurses [9]. However, concerning evidence of and guidelines on the frequency of vital sign monitoring in PACUs and the impact of such documentation on outcomes, Zeitz and McCutcheon found that the frequency of vital sign collection in the first 24 h after surgery—including the time spent in the PACU—was often routine-based [10]. We found only one reference about frequency for vital sign monitoring in the PACU within the literature: a recommendation to record vital signs as often as necessary, but at least every 15 min, during the patient’s time in the PACU [11]. The technical limitations of monitoring and compliance difficulties when it comes to very young children (aged 0–36 months) are particularly challenging. Nurses assigned to the PACU must therefore possess and utilise critical care thinking skills and, for optimal patient safety and outcome, remain vigilant for the slightest change in patient status.

There is a lack of research on the experiences of critical care nurses (CCNs) and registered nurse anaesthetists (RNAs) in providing postoperative care to children aged 0–36 months and making decisions regarding vital sign monitoring. This paper aims to address this gap in the literature. We aimed to explore the perceptions of critical care nurses and registered nurse anaesthetists regarding their experiences and actions when making decisions regarding vital sign monitoring of children in post-anaesthesia care units.

## 2. Materials and Methods

### 2.1. Design

This study employs an inductive, retrospective, qualitatively descriptive design utilizing the critical incident technique. This method was selected for its suitability in understanding the complexity of organizational structures and human interactions [12,13]. The critical incident technique is a process for collecting key details of human behaviour during specific, well-defined incidents of significant relevance, termed critical incidents [13]. These incidents are characterized by substantial positive or negative outcomes [12]. An incident is defined as critical when it is crucial in terms of its consequences and effects. Thus, the critical incident technique involves exploring and identifying participants’ experiences during critical incidents and the behaviours, activities, or actions they use to manage the phenomenon in question. This technique was considered appropriate for the present study due to its aim of exploring CCNs’ and RNAs’ experiences and actions when making decisions related to the vital sign monitoring of children in the PACU. The study was conducted in adherence with the Consolidated Criteria for Reporting Qualitative Research (COREQ) [14].

### 2.2. Setting and Participants

The study was conducted at two hospitals with PACUs, both of which provide advanced postoperative care for children. One PACU was located at a children’s hospital and cared for children who had undergone minor and major surgeries. The other PACU was at a hospital for both children and adults and cared for children who had undergone head and neck surgery and radiological examinations.

Nurses were recruited from two PACUs located in different hospitals in Sweden. The participants in this study (n = 17) were CCNs and/or RNAs. They were educated registered nurses (RNs); in Sweden, becoming an RN requires 3 years of undergraduate-level higher education with 180 credits, including a Bachelor of Science in Nursing. To become a CCN or RNA, 1 year of study in graduate-level higher education is required, which includes a postgraduate diploma in specialist nursing and a 1-year master’s degree (60 credits) [15]. As described above, the interviewed nurses worked at either a children’s hospital PACU or a PACU that cares for both adults and children. In this context, their PACU work involved working autonomously and providing an advanced level of nursing care. Both PACUs were open for 24 h a day during the studied time period. At the child’s hospital, the RNAs in the PACU also worked in the operating theatre and had typical duties for RNAs. Table 1 provides the sociodemographic and professional characteristics of the interviewed CCNs and RNAs.

Purposeful sampling was used to ensure the recruitment of CCNs and RNAs from the two settings who had experience with monitoring children aged 0–36 months—more specifically, infants (0–1 years) and toddlers (1–3 years)—in the PACU. At each PACU, the head of the department was contacted and asked to identify individuals appropriate for the inclusion criteria who might be willing to participate in the study. Invited nurses who agreed to participate in the study were telephoned and given further oral and written information on the study. Written information on the study was then sent to the invited nurses’ homes. The nurses were invited to read the information and given time to decide on whether to participate or not. They were then contacted again, and face-to-face interviews were arranged for prospective participants, who gave their consent in writing before the interviews started.

### 2.3. Data Collection

Individual semi-structured interviews were held using an interview guide from Fridlund [13]. In the interviews, the participants were asked to provide a description of a complete critical incident. Prior to data collection, two pilot interviews were conducted. The questions were then adapted to the situation and context, with the pilot interviews being excluded from the results. A total of 17 individual semi-structured interviews were held during the spring 2022: one on Zoom and the other 16 at the interviewees’ workplace, in privacy, during working hours. The interviews were conducted by four master’s students under the supervision of the first author. All sound was recorded using an external device and transcribed verbatim. The participants were informed in advance that the interviews would focus on critical incidents that involved decision-making on the monitoring of children in the PACU. The opening questions were: (a) “Can you tell me about a situation when a child’s vital signs were being monitored postoperatively, after paediatric surgery, and you perceived good decision-making about the extent of the monitoring?” and (b) “Can you tell me about a situation when you perceived poor decision-making about the extent of the monitoring?” The duration of the interviews ranged from 10 to 28 min. The authors agreed that data saturation was reached when the number of critical incidents was ‘satisfactory’, in the sense that the interviewees were beginning to repeat remarks that had been made in previous interviews. In line with the method, the number of incidents is more important than the number of interviews; 100 incidents are judged to be sufficient to reach methodological rigour, but if the incidents are complex, more incidents may be needed to reach data saturation [12].

### 2.4. Data Analysis

The data analysis, which was performed in accordance with the critical incident technique, started with a reading of the transcripts from the interviews to gain familiarity with the content. Critical incidents related to the aim were identified and picked out as illustrating either good or poor decision-making. Initially, the transcripts from the two PACUs were analysed separately. It appeared that similar critical incidents occurred regardless of which PACU was involved, and it was judged to be irrelevant to compare the analyses. This was followed by a structural analysis using critical incident technique [12]. Meaningful experiences and actions were extracted from the data, labelled and compared in terms of similarities and differences for grouping in subcategories. The subcategories were then labelled, and corresponding comparisons among them were carried out to sort them into categories. From the categories, the main areas relating to experiences and actions emerged [12,13]. The first author was mainly responsible for the analysis; however, discussions among all the authors took place throughout the process until consensus was reached on the definitive main areas, categories, and subcategories.

### 2.5. Ethics

Ethical approval was granted by the Institutional of Swedish Ethical Review Authority (ref. 2022-00896-01). The study complies with the Helsinki Declaration and all informants have been informed and consented to the study.

## 3. Result

The CCNs’ and RNAs’ references to decision-making regarding the children’s need for vital sign monitoring involved electrocardiograms (ECG)s, pulse oximetry, and invasive and non-invasive blood pressure measurement. The nurses’ perceptions of their experiences and actions emerged in 241 critical incidents. Two main areas emerged in the participants’ experience of monitoring children in PACUs: adequate and inadequate grounds for decisions. Within these two areas, there were nine categories and subcategories (Table 2). One main area emerged in the participants’ actions in children’s monitoring: adjusting of the monitoring. Within this area, there were three categories and subcategories (Table 3).

### 3.1. Adequate Grounds for Decisions

#### 3.1.1. Prescriptions and Guidelines

The CCNs and RNAs followed the guidelines closely for every child. In addition, they engaged in a dialogue on adherence to prescriptions and guidelines with the physician in charge and with health professionals in other units who were involved in the child’s care. The guidelines provided support when difficulties arose, a child was disturbed by the monitoring, or the children’s parents and/or the nurses’ colleagues asked questions.

#### 3.1.2. Potential of Technology

The CCNs and RNAs prioritised pulse oximetry; they attempted to maintain it when the children were recovering from anaesthesia. Pulse oximetry was described as valuable for monitoring heart rate, detecting arrhythmias, and monitoring a child’s airway and breathing. Moreover, when children needed opioids, sedatives, and/or oxygen therapy during their recovery, pulse oximetry was crucial in monitoring and evaluation. The primary purpose of ECG monitoring was to detect arrhythmias; however, seeing the heart rate on the monitor was valuable, since it can be difficult to find a pulse on a young child. Moreover, when a child wakes up, the pulse may rise from 140 to 180 beats per minute. The monitor display of a child’s respiratory rate when an ECG is in progress was found to be especially useful in supplementing the clinical assessment.

#### 3.1.3. Clinical Assessment

Experience of and skill in clinical assessment were described as having a major impact on decisions about children’s monitoring in the PACU. These qualities gave the nurses the intuition to sense impending changes in a child’s condition. The PACU at the children’s hospital was staffed by the same RNAs working in the operating theatre. These RNAs considered this overlap to be a positive determining factor in their work and their decisions about the monitoring of children in the PACU.

#### 3.1.4. The Child’s Health Status

Aside from the guidelines, decisions on monitoring children during their recovery in the PACU were based primarily on what had happened in the intraoperative period. Decision-making also involved an individualised, child-centred approach to monitoring. For example, the monitoring was adapted to children with special needs, children who (owing to disease) frequently returned to the PACU, and unique situations that might affect any child.

#### 3.1.5. Participating Parents

The participants emphasised the importance of involving the parents during a child’s recovery. The involved parent was a resource for the child in an unfamiliar environment, and the parents’ closeness to their children enabled the parents to help recognise signs through the child’s voice and smell. Parents know their children, and the CCNs and RNAs described parents as an asset, as parents can notice changes in their children and confirm the child’s usual status.

#### 3.1.6. Situational Awareness

The participants were aware of the importance of being prepared for uncommon, complex and/or acute situations in relation to decisions on monitoring children. The CCNs and RNAs emphasised the importance of remaining calm in such situations and trusting their own clinical assessment.

### 3.2. Inadequate Grounds for Decisions

#### 3.2.1. Limits of Technology

The CCNs and RNAs were aware of technical limitations. In their experience, they were able to see changes in the child clinically before they showed up on the monitor. The pulse oximeter is often fitted peripherally on a child’s small fingers, where the circulation is poor; this makes measuring more difficult and gives rise to low values. Using an ECG to monitor breathing means that changes in breathing are often first observed clinically. Overall, using these technologies naturally implies a delayed alarm function. It also means that—in order to serve as a reliable basis for assessment—assessment through monitoring must be prolonged.

In general, the participants found it difficult to obtain reliable values from all types of monitoring when children were recovering from anaesthesia, becoming alert, and starting to move. Pain, hunger, anxiety, and the unfamiliar situation were other reasons for errors in measuring the children’s vital signs at this time. Non-invasive measurement of babies’ blood pressure is particularly difficult: it stresses them, makes them agitated, and leads to decisions based on incorrect figures. Incorrect positioning of the equipment may be another cause of incorrect readings. Both the pulse oximeter and the ECG electrode can become stuck on the child’s blanket.

#### 3.2.2. Inappropriate Use of Technology

Technology usage that is contrary to the accepted guidelines may occur in the PACU because decision-making is a matter of balancing what the monitoring adds to the assessment of the child against the discomfort that monitoring may entail, which in turn may lead to other problems for the child. Non-invasive blood pressure monitoring is an example of this kind of measure; thus, careful consideration must take place before and while blood pressure is measured. When CCNs and RNAs evade non-invasive blood pressure monitoring they trying to avoid a situation when a child become so agitated that bleeding can occur from the injection sites and surgical wounds. Another opposed example involves not taking the opportunity to use the ECG to monitor breathing, which would enable any impairment to be detected earlier. Moreover, children with heart failure may have a lower saturation level in their habitual state. In such cases, it is important to adjust the alarm limits to the child’s habitual values in order to avoid incorrectly based decisions.

#### 3.2.3. Decisions Made by a Previous Doctor or Nurse

CCNs and RNAs in the PACU commonly encounter situations in which decision-making on the level of a child’s postoperative monitoring has been made perioperatively. Even at the children’s hospital, where the RNAs in the PACU also worked in the operating theatre, the RNAs did not always agree with their colleagues’ decisions. The nurses’ perception was that it is easier to discontinue unwanted monitoring than to commence it if desired. On other occasions, when disagreements arose, these involved what the nurses regarded as the wrong level of care being given to children who needed more resources than were available. Such a situation could occur when an ordinary unit with more resources closed, causing a child’s care and treatment there to be transferred to the CCNs and RNAs in the PACU.

### 3.3. Adjusting the Monitoring

#### 3.3.1. Reducing the Interference Caused by Monitoring

The nurses found that monitoring vexed the children who were under observation in the PACU. To prevent the child having the operation from becoming agitated and to avoid a situation in which the child might pull out peripheral and central venous catheters, the health professionals in the operating theatre removed as much of the monitoring equipment as possible before the child arrived in the PACU. When a child seemed to be disturbed by the monitoring, as much of the equipment as possible had to be removed. One situation that was decisive in deciding to discontinue the monitoring was when the child, held in the parent’s arms, was trying to feed. At such a time, the monitoring equipment was a hindrance, and the most typical decision was that the child was sufficiently wide awake to no longer require monitoring. The CCNs and RNAs were unanimous in their view that, if everything looked fine, children should not be touched in any way on their arrival in the PACU. In other words, the children should be allowed to remain under anaesthesia and come out of it naturally, when they were ready. The interviewees expressed the hope that the children would then wake up in a tranquil state.

#### 3.3.2. Monitoring Based on the Current Situation

However, depending on what happened during a child’s perioperative period and recovery in the PACU, expanded monitoring might be required. This decision could be based on the clinical assessment. It was also common for monitoring to recommence, often when children received painkillers or sedatives. Another means of adjusting the monitoring to align with the child’s state was to take measurements intermittently—mostly with a pulse oximeter—or to leave the blood pressure cuff on but unplugged and then connect the cord when the opportunity to measure arose. When there is a heavy workload in the PACU, a high level of machine-based monitoring is often necessary, as the nurses cannot provide enough personal attention to each patient. At such times, the CCNs and RNAs did not have the same opportunity to be close to the children and observe them; thus, they were more cautious in their decisions to discontinue the monitoring. Moreover, during the night shift, they exercised additional caution before disconnecting the monitoring equipment. Sometimes they left it connected and let the receiving specialist nurse make the decision. On the other hand, if the child had good readings on the pulse oximeter curve, experienced CCNs and RNAs might decide to stop the monitoring. Moreover, since faulty alarm signals from the monitors can be very disturbing for children and parents, alarm settings sometimes were adapted with reduced sound level. On these occasions, the CCNs and RNAs increased their observations of and presence near to the children and used their clinical judgement. If they disconnected the monitoring, they did so because it added nothing to their assessment and might make the parents overly anxious, even when the specialist nurses pointed out that the alarm was in error.

#### 3.3.3. Non-Medical Technical Assessment

As part of their clinical assessment, the CCNs and RNAs observed the children’s breathing patterns. All the nurses’ senses were used in their overall assessment: they listened, looked and felt to assess the child’s skin tone, sweat on the forehead, facial expressions, stiffness, motor skills, and capillary refill. Depending on the specific surgical intervention that had been performed on a child, the participants were aware of the various complications that might arise during the child’s recovery. The nurses described being close to the child’s bedside all the time in order to immediately detect changes in the child’s condition, especially if no monitoring was taking place. The children’s parents were also important; some of the participants used the parents to aid in their assessment or to help divert the child so that the nurses could reconnect the apparatus.

## 4. Discussion

When monitoring a child’s vital signs to ensure safe recovery, the CCNs and RNAs found that decision-making required them to constantly be near the child and to adjust the level of monitoring and find other ways to achieve the necessary level of monitoring other options, as intermittent measures were necessary. Uniform recovery assessment involves several difficulties. The guidelines emphasise the importance of reliable vital sign monitoring to indicate children’s current physiological status in the PACU [7]. The most common complications were respiratory; risk factors had to be considered and optimised before the children’s surgery, with a purpose and a plan for vital sign monitoring in order to promote safe recovery from anaesthesia and surgery [16]. However, the interviewed nurses found that implementing such a plan was problematic, with one reason being the absence of guidelines about the frequency of vital sign monitoring [10]. The transfer of children from the operating theatre to the PACU can be a critical stage, since adverse events tend to occur during the child’s first 30 min in the PACU. Thus, it is essential for the CCNs and RNAs working in PACUs to be skilled in advanced airway management [17]. At the children’s hospital, decision-making on monitoring vital signs in the PACU was primarily based on local guidelines; the child’s age, ASA classification, and state of ill-health, as well as the events that occurred during the intraoperative period. As mentioned earlier, this PACU was staffed by the same RNAs who worked in the operating theatre. Nonetheless, the RNAs did not always agree with their colleagues’ decisions about the level of monitoring required. The nursing literature confirms this finding and illustrates the complexity of how experience best benefits acute medical decision-making [18]. The CCNs and RNAs used a combination of clinical assessment and the monitoring of vital signs to assess the children’s recovery. Their perception was that experience and ability in clinical assessment had a major impact on decisions regarding the children’s need for monitoring, serving as grounds for decision-making. This perspective is well known but controversial. However, an integrative review has shown that intuition plays a role in clinical decisions, alongside research-based evidence, supporting a nursing process based on knowledge and care experience—a basis for decision-making that promotes safe patient care [19]. For example, the NEWS 2 scoring system incorporates evidence into nursing practice; in addition to the assessment, it emphasises that nurses need not wait for the patient to ‘trigger’ and should escalate their concerns before they act [20].

The nurses’ assessment could also involve the parents, whose knowledge of their children made them an asset, in the CCNs’ and RNAs’ view. This might have been problematic, however, if the parents did not really want to be involved. To provide compassionate postoperative care for a young patient, it is vital for nurses to be aware of the parents’ experience and need for involvement, which may vary [21]. It is common for the professionals at a hospital to have differing opinions on whether parents should evaluate the child’s status in the PACU. The CCNs and RNAs experienced technical limitations and difficulties in convincing the children to accept vital sign monitoring. In general, the participants thought that it was difficult to obtain reliable measurements from all types of monitoring for children that are recovering from anaesthesia. Although wireless solutions (e.g., camera-based techniques to monitor vital signs) have been used in neonatal care, camera settings and environmental parameters influence accuracy [22]. Therefore, it is probable that this technique will not contribute to the monitoring of vital signs in paediatric postoperative care.

In general, children undergoing surgery are healthier than adults. However, as young children have smaller reserves and immature organs, there are limits to their ability to compensate for the complications that may arise during the perioperative period [23]. Of course, another key factor in the risk of postoperative adverse events is the child’s classification according to the ASA guidelines. The CCNs and RNAs related how they used strategies such as situational awareness to be prepared and to reduce postoperative adverse events. Tower [24] considers nurses’ situational awareness to include perceptions about patients’ progress during their hospital stay. This report aligns with the perceptions of the participants in this study, who found that experience makes it possible to foresee changes in a child’s condition. The CCNs and RNAs described clinical assessment of the child’s breathing as crucial. The nurses were constantly near the child’s bedside in order to immediately detect changes in the child’s condition, especially if the child had rejected the monitoring and it had been discontinued. The interviewed CCNs and RNAs pointed out the importance of the monitoring and how valuable it was to them when clinical assessment was difficult and subject to limitations. Pulse oximetry was considered to be the most valuable technique, and it was the one most used by the participants. In addition to pulse oximetry, capnography may afford an opportunity to detect respiratory adverse events earlier and thus reduce their degree of severity. However, additional research is needed and RCTs are necessary, as McNeill and Tabet discuss in their review [25]. Further problems for young children are related to their tolerance of the monitoring and the risk of acute agitation, which is common after anaesthesia. This may also cause great anxiety for the patient, family and caregivers, and make them even more concerned for the child’s physical safety [26]. Therefore, the CCNs and RNAs made adjustments, depending on the children and their current situation, to achieve an individualised, child-centred approach in each child’s monitoring.

To boost reliability, confirmability, and transferability, and to reduce influence on the authors’ pre-understanding in this study, the authors followed the guidelines and five steps of critical incident technique carefully [12,13]. The first step involved identifying an aim that could be achieved via an analysis of the CCNs’ and RNAs’ skills and knowledge in the given context. For the second step, which covers the specification of the types of incidents, Flanagan has recommended that the incidents should be remarkably effective or ineffective—that is, they should make a major difference [12]. This criterion was applicable in the context of this study, in which the CCNs’ and RNAs’ actions stemmed from a deep understanding of the whole situation. According to Flanagan, the selected participants must be the ’right’ people, whose actions make a significant difference in the incident and thus whose ’reflection in action’ holds valuable information for the study [12]. In step three, we collected the data using Fridlund interview guidelines, which resulted in 241 critical incidents. If a phenomenon is ‘significantly delimited’, Flanagan states that 50–100 incidents are enough [12]. In step four, we analysed the data through interactive discussions with the research team, whose members had varying levels of pre-understanding and knowledge of the study context. Finally, in step five, we presented the results by describing, explaining, comparing, reflecting on, and ultimately exploring their implications [13]. The data provide a rich description of the participants’ experiences and the situations in which these experiences occurred [13].

For the transferability of the result the authors have made efforts to describe the participating CCNs and RNAs, context, data collection and analysis as carefully as possible.

Nevertheless, this study has some limitations. The purposeful selection of participants resulted in a group characterised by long professional experience and advanced age. The participants well represented the nurses working in the selected PACUs, and no greater variation in age and professional experience was possible. However, in a study like this, the results invariably depend on how well the informants remember and can describe the incidents in question, as well as the interviewer’s ability to prompt the informants to describe them.

## 5. Conclusions

The findings of this multicentre study indicate that providing postoperative care to children aged 0–36 months necessitates addressing both appropriate and inappropriate rationales for vital sign monitoring. While the technology is valuable when used alongside clinical assessment, it can pose risks if the child does not tolerate the monitoring. Therefore, knowledge, experience, proper management, and an understanding of the significance of vital sign monitoring are crucial for nurses in the PACU. Various strategies are employed to adjust monitoring during children’s recovery to optimize conditions for vital sign monitoring in the current context. The complexity of accurately monitoring children presents challenges for nurses in the PACU to adhere to guidelines. Evidence-based care and safety are compromised when technology has limitations and is not tailored for paediatric use, resulting in a greater reliance on experience and clinical assessment. This not only underscores the importance of experience for reliable assessment but also indicates that greater risks are being accepted.

## Figures and Tables

**Table 1:** Sociodemographic and professional characteristics of the interviewed CCNs and RNAs (n = 17).

	Median	Range
Age, years	53	30 to >60
Number of years of healthcare experience	34	10 to >30
Number of years as trained specialist nurse	20	1 to >30
Department		
Working in PACU at children’s hospital		9
Working in PACU with both adults and children		8

**Table 2:** Summary of subcategories, categories, and main areas of the CCNs’ and RNAs’ experiences.

Subcategories	Category	Main Area
Standard monitoring for various interventions (10) * Routines followed (8) Dialogue/prescription with doctor or nurse in charge (8)	Prescriptions and guidelines	Adequate grounds for decisions
ECG connection providing graph of breathing (6) ECG used for heart rate and rhythm (8) Pulse oximetry as a technical aid (13)	Potential of technology	
Experience is important (14) Sense of impending change for the worse (11) Transfer to nurse anaesthetists from operation staff for recovery from anaesthesia (4)	Clinical assessment	
Adaptation to what happened in the intraoperative period (6) Child-centred approach and individualisation (15) Child’s age (5) ASA class, state of ill-health (5)	Child’s health status	
Parents know their children, are close by, provide security and/or perceive change promptly (8)	Participating parents	
Need to be prepared for unusual and/or complex emergency situations (7) Keeping calm in critical situations (2)	Situational awareness	
Delay in readings displayed if child’s condition worsens (3) Positioning may result in incorrect readings (5) Agitated child, pain, hunger, restlessness and/or strange situation causing incorrect readings (4) Difficult to measure blood pressure reliably in very young children (3) Technology must be used at more frequent intervals for correct assessment (1)	Limits of technology	Inadequate grounds for decisions
Existing scope for monitoring not used (1) Child’s ill-health means that lower saturation level is important; correction of alarm thresholds necessary (1)	Inappropriate use of technology	
Decision on level of monitoring made intraoperatively (3) Wrong level of care; child who needs more resources than are available (4) Regular unit closes and child has to be transferred from there (1)	Decisions Made by a Previous Doctor or Nurse	

* Number of incidents in brackets.

**Table 3:** Summary of quotations, subcategories, categories and main areas of the CCNs’ and RNAs’ actions.

Quotations	Subcategories	Category	Main Area
“So we disconnect it when they come in from the operation, or we ask the anaesthetic staff not to keep doing the ECGs and blood pressure monitoring. We don’t have it on any children”.	Remove monitoring in advance (1) *	Reducing interference caused by monitoring	Adjusting the monitoring
“When they get so irritated and cross, and scream and just thrash their feet around, because then there’s a risk of them pulling out the peripheral venous catheters and everything that entails, or the central venous catheters as well, so that you really have to remove the pulse oximeter”.	Remove monitoring (7)		
“We have extremely little monitoring—we take off what we can”.	Remove as much as possible (3)		
“If they’ve woken up and had something to eat, then they don’t need monitoring any more anyway, generally speaking”.	Remove monitoring when they (the child) are picked up and held (10)		
“Because we don’t want to disturb them. They need to get plenty of sleep after having their anaesthetic, with all the medication. If they wake up too early, we get a real uproar here, you see. They toss around, and they’re just not themselves”.	Keep monitoring (8)		
“Where something has happened during the operation that results in the child needing to be monitored more clearly”.	Extended monitoring (1)	Monitoring based on the current situation	
“Usually once you’ve administered the morphine, they calm down and then you can put the pulse oximeter back again”.	Option of reconnecting the equipment (6)		
“And then you have to, sort of, just sneak it on for a short while, and then you can take it away again, for instance. Then all you can do is hold still for a couple of seconds and say ‘finished’, and then we take it off, and so it goes on”.	Intermittent checks (7)		
“On the other hand, if you were to have masses of children at the PACU on some occasion, that you don’t really have a chance to look at them that much—then perhaps you should be more cautious about removing the monitoring”.	Keep the monitoring equipment on when there are a lot of children in the PACU and/or at night (1)		
“Then we send everything with the child to the department as well. So then they can decide when to remove it, because it’s much easier to remove the stuff than to put it back again”.	Keep the monitoring on when the child is sent to the department (1)		
“Is it worth waking up the child so as to, perhaps, then take an extra blood pressure reading? Or should we let the child sleep?”	Refrain from monitoring (2)		
“And then we ask the doctor whether it’s OK for us just to use a pulse oximeter in here”.	Doctor’s prescription (6)		
“Yes, that’s how it is: having had long experience, I’ve learnt a thing or two about what can happen and what should happen. So I don’t always contact the doctor every time”.	Personal decision (4)		
“Then you turn them off. You have to be there anyway, keeping an eye on them anyway, all the time”.	Change the alarm limits (4)		
“You also do a tremendous lot of watching how the children are breathing, and of course check whether the alae nasi are moving or retracted, whether there’s stridor, and that sort of thing”.	Breathing patterns (7)	Non-medical technical assessment	
“I watch the child closely, you could say. I touch the child and look, without staring, to see the skin tone, sweat on the forehead, mimicry, rigidity, how the hands are opening and shutting, I take the hands and feel whether they’re tense, and I watch and listen to the breathing”.	Use one’s own senses in the observation (9)		
“Then I may skip the monitoring and just look at them. But then you really look at them—then you can’t sit at a distance, in front of the computer or anything like that. You really have to be close by if you don’t have any monitoring on at all”.	Near the child (4)		
“You have another person there, after all, but you absolutely mustn’t ask too much of the parents. You’ve got one other person who knows them and who knows about what’s normal and what isn’t”.	Get the parents involved (4)		

* Number of incidents in brackets.

## Data Availability

The qualitative data upon which this analysis was conducted are not publicly available due to ethical concerns regarding confidentiality of participants. Further, consent was not obtained from participants to share information from interview transcripts with third parties not involved in the research, and the ethical approval for this study does not permit the sharing of such information.
